# COVID-19 and its Cardiac and Neurological Complications among Ontario Visible Minorities

**DOI:** 10.1017/cjn.2021.148

**Published:** 2021-06-24

**Authors:** Joseph Y. Chu, Yosuf Kaliwal, Maria Koh, Robert Chen, Chi-Ming Chow, Dennis T. Ko, Peter P. Liu, Gordon W. Moe

**Affiliations:** Division of Neurology, Department of Medicine, Toronto Western Hospital-University Health Network and William Osler Health System, University of Toronto, Toronto, Canada; ICES, Toronto, Canada; Krembil Research Institute, University Health Network and Division of Neurology, Department of Medicine, University of Toronto, Toronto, Canada; Division of Cardiology, Department of Medicine, St. Michael’s Hospital, University of Toronto, Toronto, Canada; Schulich Heart Program, Sunnybrook Health Sciences Centre, Sunnybrook Research Institute, University of Toronto, Toronto, Canada; University of Ottawa Heart Institute and Department of Medicine and Cellular & Molecular Medicine, University of Ottawa and Division of Cardiology, Department of Medicine, University of Toronto, Toronto, Canada

**Keywords:** COVID-19, Cardiac complications, Neurological complications, Chinese and South Asians, Clinical epidemiology

## Abstract

**Background:**

Due to lack of data on the epidemiology, cardiac, and neurological complications among Ontario visible minorities (Chinese and South Asians) affected by coronavirus disease (COVID-19), this population-based retrospective study was undertaken to study them systematically.

**Methods:**

From January 1, 2020 to September 30, 2020 using the last name algorithm to identify Ontario Chinese and South Asians who were tested positive by PCR for COVID-19, their demographics, cardiac, and neurological complications including hospitalization and emergency visit rates were analyzed compared to the general population.

**Results:**

Chinese (N = 1,186) with COVID-19 were found to be older (mean age 50.7 years) compared to the general population (N = 42,547) (mean age 47.6 years) (*p* < 0.001), while South Asians (N = 3,459) were younger (age of 42.1 years) (*p* < 0.001). The 30-day crude rate for cardiac complications among Chinese was 169/10,000 (*p* = 0.069), while for South Asians, it was 64/10,000 (*p* = 0.008) and, for the general population, it was 112/10,000. For neurological complications, the 30-day crude rate for Chinese was 160/10,000 (*p* < 0.001); South Asians was 40/10,000 (*p* = 0.526), and general population was 48/10,000. The 30-day all-cause mortality rate was significantly higher for Chinese at 8.1% vs 5.0% for the general population (*p* < 0.001), while it was lower in South Asians at 2.1% (*p* < 0.001).

**Conclusions:**

Chinese and South Asians in Ontario affected by COVID-19 during the first wave of the pandemic were found to have a significant difference in their demographics, cardiac, and neurological outcomes.

## RÉSUMÉ

Les complications cardiaques et neurologiques liées aux infections à la COVID-19 parmi des minorités visibles en Ontario.

**Contexte:**

C’est en raison d’un manque de données portant sur l’épidémiologie ainsi que sur les complications cardiaques et neurologiques des minorités visibles de l’Ontario (de descendance chinoise ou sud-asiatique) atteintes par une infection à la COVID-19 que cette étude rétrospective basée sur la population a été entreprise en vue d’étudier cette réalité de façon systématique.

**Méthodes:**

C’est au moyen d’un algorithme des noms de famille que nous avons pu identifier des Ontariens de descendance chinoise ou sud-asiatique qui ont obtenu un résultat positif à un test PCR (*polymerase chain reaction*) de dépistage de la COVID-19, et ce, du 1^er^ janvier au 30 septembre 2020. Nous avons ensuite procédé à une analyse de leurs caractéristiques démographiques et de leurs complications cardiaques et neurologiques, ce qui inclut les taux d’hospitalisation et de visite à un service d’urgence, en comparaison avec la population générale.

**Résultats:**

Les individus de descendance chinoise (*n* = 1186) atteints de COVID-19 se sont révélés plus âgés (âge moyen = 50,7 ans) si on les compare à la population générale (*n* = 42 547; âge moyen = 47,6 ans ; *p* < 0,001) tandis que ceux de descendance sud-asiatique (*n* = 3459) étaient plus jeunes (âge moyen = 42,1 ans; *p* < 0,001). Le taux brut de complications cardiaques au bout de 30 jours parmi les Ontariens de descendance chinoise était de 169/10 000 (*p* = 0,069) alors qu’il était de 64/10 000 (*p* = 0,008) chez les Ontariens de descendance sud-asiatique et de 112/10 000 au sein de la population générale. En ce qui concerne les complications neurologiques, le taux brut de complications au bout de 30 jours était de 160/10 000 (*p* < 0,001) chez les Ontariens de descendance chinoise, de 40/10 000 (*p* = 0,526) chez ceux de descendance sud-asiatique et de 48/10 000 au sein de la population générale. Enfin, soulignons que le taux de mortalité (toutes causes confondues) au bout de 30 jours était notablement plus élevé chez les Ontariens de descendance chinoise (8,1 %) en comparaison avec la population générale (5,0 %; *p* < 0,001) alors qu’il s’est avéré inférieur chez les Ontariens de descendance sud-asiatique (2,1 %; *p* < 0,001).

**Conclusions:**

En somme, les Ontariens de descendance chinoise ou sud-asiatique touchés par une infection à la COVID-19 lors de la première vague de la pandémie ont présenté des différences significatives en ce qui regarde leurs caractéristiques démographiques et les conséquences de cette infection d’un point de vue cardiaque et d’un point de vue neurologique.

## Introduction

Severe acute respiratory syndrome coronavirus 2 (SARS-CoV-2) is responsible for the global pandemic now known as coronavirus disease (COVID-19).^1,2^ Data from outside Canada suggest that there are substantial disparities among different ethnic groups in who gets infected and who have adverse outcomes.^14,17,18,28,29^ In the USA, according to a report by Center for Disease Control in 2020,^13^ 33% of hospitalized patients were black, compared to 18% in the community, and 8% were Hispanic, compared to 14% in the community. The overall mortality rates among African Americans were much higher than that of the white and Asian persons.^13^ However, the racial distribution in the impact of COVID-19 in Canada has not been studied systematically.^2,10,19^ In Ontario and based on the 2016 Canadian census, Chinese accounts for 19.4%, while South Asians accounts for 29.6% of visible minorities.^42^ Therefore, these two ethnic groups account for almost 50% of visible minorities in Ontario. Despite the collected data on COVID-19 in the general population of Ontario,^1^ however there is a paucity of information on how these two minority ethnic groups are affected by COVID-19, in particular their rates and types of cardiac and neurological complications. Accordingly, we conducted a population-based retrospective study to evaluate the epidemiology as well as potential cardiac^31,33,34^ and neurological complications^4,5,43,44^ of COVID-19 among Chinese and South Asians compared to the general population in Ontario.

## Methods

### Data Sources

To identify the cohort, we used the Ontario Laboratory Information System database and selected those who had a positive COVID test result between January 1, 2020 and September 30, 2020 inclusive. To identify baseline demographics and health conditions, health outcomes, and health service use, we used data from the Registered Persons Database (RPDB), Canadian Institute for Health Information (CIHI) Discharge Abstract Database (DAD), the National Ambulatory Care Reporting System (NACRS), and the Ontario Health Insurance Plan (OHIP) physician claims database. The RPDB provides basic demographic information about anyone who has ever received an Ontario health card number. The DAD contains patient-level data for hospitalizations. The NACRS captures information on patient visits to emergency departments. OHIP captures information of the services provided by practicing physicians in Ontario. These data sets were linked using unique encoded identifiers and analyzed at ICES.

### Study Population

The cohort included individuals between the ages of 18 and 105, who had a positive COVID-19 test between January 1, 2020 and September 30, 2020. We excluded patients who were not Ontario residents at the time of the COVID-19 test. If the person had multiple positive tests within the study period, the first positive test date was chosen as the index date. Individuals’ ethnicities (classified into Chinese, South Asian, and all others hereby termed General) were determined using an algorithm developed by Shah et al. which uses a surname-based approach to identify ethnicity based on an individuals’ surname.^40^ The use of the data in this project is authorized under section 45 of Ontario’s Personal Health Information Protection Act (PHIPA) and does not require review by a Research Ethics Board.

### Outcomes

The primary outcome of interest was death. Cardiac outcome including myocardial infarction, heart failure, arrhythmia, atrial fibrillation and flutter, myocarditis, deep vein thrombosis/pulmonary embolism (DVT/PE). Neurological outcomes include hemorrhagic stroke, ischemic stroke, seizure, meningitis, encephalitis, encephalopathy, and Parkinson’s disease. Health service use includes hospitalization, emergency room visit, intensive care units (ICU) admission, use of extracorporeal membrane oxygenation, and use of mechanical ventilation within 30 days of having a positive COVID-19 test (Codes are listed in Appendix A). The outcomes were measured at the individual level indicating whether the patient did or did not experience the outcome.

### Statistical Analysis

Baseline and outcome characteristics were compared between the Chinese and South Asians versus the general population. In addition, Charlson comorbidity index and 30-day all-cause mortality rate, cardiac and neurological complication rates were also analyzed. For continuous variables, descriptive statistics included mean values with standard deviation, median values with interquartile range, and the *p*-values were calculated using one-way analysis of variance for means and Kruskal–Wallis test for medians. Categorical variables were described using proportions, and *p*-values from a chi-squared test were provided. *p*-Values were used to compare the Chinese population to the general population and to compare the South Asians population to the general population. Multivariable logistic regression was used to determine if ethnicity was associated with death, cardiac outcomes, neurological outcomes, or hospitalization or emergency department visits. A separate logistic regression model was built for each binary outcome with ethnicity as the main exposure categorical variable and the general population as the reference group. Adjustment variables included age, sex, income quintile which is defined as the quintile of neighborhood income per person equivalent within a census metropolitan area, census agglomeration or residual area (Table 1), and long-term care placement within 90 days (Table 2) prior to positive COVID test. Odds ratios (ORs) comparing Chinese and South Asian ethnicities to the general population were computed for each outcome.

**Table 1:** Baseline characteristics of patients by ethnic group

Characteristics	Chinese	South Asian	General	Overall	*p*-Values	*p*-Values
	N = 1,186	N = 3,459	N = 42,547	N = 47,192	Chinese vs General	South Asian vs General
Age, mean (SD)	50.7 ± 21.9	42.1 ± 19.2	47.6 ± 23.0	47.3 ± 22.8	<.001	<.001
Age, median (IQR)	51 (32–64)	40 (27–56)	46 (28–63)	46 (28–62)	<.001	<.001
Age groups					<.001	<.001
19 and under	58 (4.9%)	320 (9.3%)	3,737 (8.8%)	4,115 (8.7%)		
20 to 39	355 (29.9%)	1,402 (40.5%)	13,830 (32.5%)	15,587 (33.0%)		
40 to 59	403 (34.0%)	1,059 (30.6%)	12,479 (29.3%)	13,941 (29.5%)		
60 to 79	204 (17.2%)	546 (15.8%)	7,208 (16.9%)	7,958 (16.9%)		
80+	166 (14.0%)	132 (3.8%)	5,293 (12.4%)	5,591 (11.8%)		
Sex					0.808	<.001
Male	546 (46.0%)	1,865 (53.9%)	19,739 (46.4%)	22,150 (46.9%)		
Female	640 (54.0%)	1,594 (46.1%)	22,808 (53.6%)	25,042 (53.1%)		
Income Quintile					0.365	<.001
Income Quintile 1 (lowest)	335 (28.2%)	659 (19.1%)	12,066 (28.4%)	13,060 (27.7%)		
Income Quintile 2	268 (22.6%)	935 (27.0%)	8,990 (21.1%)	10,193 (21.6%)		
Income Quintile 3	205 (17.3%)	1,012 (29.3%)	8,340 (19.6%)	9,557 (20.3%)		
Income Quintile 4	194 (16.4%)	560 (16.2%)	6,778 (15.9%)	7,532 (16.0%)		
Income Quintile 5 (highest)	181 (15.3%)	284 (8.2%)	6,205 (14.6%)	6,670 (14.1%)		
Residence					<.001	<.001
Rural	6 (0.5%)	15 (0.4%)	1,528 (3.6%)	1,549 (3.3%)		
Urban	1,177 (99.2%)	3,435 (99.3%)	40,867 (96.1%)	45,479 (96.4%)		

**Table 2:** Baseline characteristics of patients by ethnic group: comorbidities, long-term care status, and hospitalization

Characteristics	Chinese	South Asian	General	Overall	*p*-Values	*p*-Values
	N = 1,186	N = 3,459	N = 42,547	N = 47,192	Chinese vs General	South Asian vs General
Asthma	135 (11.4%)	542 (15.7%)	6,823 (16.0%)	7,500 (15.9%)	<.001	0.571
Diabetes	209 (17.6%)	683 (19.7%)	7,302 (17.2%)	8,194 (17.4%)	0.679	<.001
Hypertension	389 (32.8%)	884 (25.6%)	12,987 (30.5%)	14,260 (30.2%)	0.093	<.001
Heart failure	40 (3.4%)	93 (2.7%)	2,243 (5.3%)	2,376 (5.0%)	0.004	<.001
COPD	23 (1.9%)	43 (1.2%)	1,521 (3.6%)	1,587 (3.4%)	0.003	<.001
Dementia	140 (11.8%)	107 (3.1%)	4,881 (11.5%)	5,128 (10.9%)	0.723	<.001
Chronic kidney disease	55 (4.6%)	100 (2.9%)	2,159 (5.1%)	2,314 (4.9%)	0.498	<.001
LTC within 90 days from testing date	162 (13.7%)	110 (3.2%)	5,207 (12.2%)	5,479 (11.6%)	0.141	<.001
LTC status on testing date	76 (6.4%)	48 (1.4%)	2,136 (5.0%)	2,260 (4.8%)	0.031	<.001
Hospitalization (last 5 years from index)	317 (26.7%)	696 (20.1%)	12,054 (28.3%)	13,067 (27.7%)	0.227	<.001

COPD = chronic obstructive pulmonary disease, LTC = long-term care.

## Results

Chinese (N = 1,186) infected by COVID-19 were older with a mean age of 50.7 years compared to general population (N = 42,547) of 47.6 years (*p* < 0.001), while South Asians (N = 3.459) were younger with a mean age of 42.1 years (*p* < 0.001). 14.0% of Chinese and only 3.8% of South Asians were >80 years compared to 12.4% of the general population (Table 1). There was no statistical difference in sex distribution for Chinese, while for South Asians 53.9% were male compared to 46.4% in the general population (*p* < 0.001). Income quintile showed that only 8.2% of South Asians were in the highest category (quintile 5), while for general population, it was 14.6%. The majority of both Chinese and South Asians (99.2% and 99.3%) and the general population (96.1%) were from urban areas (Table 1). Among the components of the Charlson comorbidity index (past 5 years from index), for Chinese, only chronic obstructive pulmonary disease or other respiratory diseases (1.0%) vs general population (2.3%) were significantly different (*p* = 0.004) (Table 3). Rate of past hospitalization was similar in Chinese (26.7% vs 28.3%, *p* = 0.227), while it was lower among South Asians (20.1% vs 28.3%, *p* < 0.001) (Table 2).

**Table 3:** Baseline characteristics of patients by ethnic group: Charlson comorbidity index

Characteristics	Chinese	South Asian	General	Overall	*p*-Value	p-Value
	N = 1,186	N = 3,459	N = 42,547	N = 47,192	Chinese vs General	South Asian vs General
Charlson comorbidity index (past 5 years from index)						
Acute Myocardial Infarction	14 (1.2%)	32 (0.9%)	515 (1.2%)	561 (1.2%)	0.926	0.137
Congestive Heart Failure	27 (2.3%)	46 (1.3%)	1,123 (2.6%)	1,196 (2.5%)	0.441	<.001
Peripheral Vascular Disease	8 (0.7%)	8 (0.2%)	309 (0.7%)	325 (0.7%)	0.836	<.001
Cerebrovascular Disease	33 (2.8%)	32 (0.9%)	963 (2.3%)	1,028 (2.2%)	0.237	<.001
Dementia	62 (5.2%)	48 (1.4%)	2,136 (5.0%)	2,246 (4.8%)	0.747	<.001
Chronic Obstructive Pulmonary Disease or other Respiratory diseases	12 (1.0%)	27 (0.8%)	965 (2.3%)	1,004 (2.1%)	0.004	<.001
Rheumatic-like Diseases	≤5	≤5	119 (0.3%)	124 (0.3%)	0.365	0.002
Ulcers of the Digestive System	7 (0.6%)	11 (0.3%)	230 (0.5%)	248 (0.5%)	0.818	0.081
Liver Disease - Mild	≤5	≤5	150 (0.4%)	158 (0.3%)	0.382	0.004
Diabetes - No Chronic Complications	41 (3.5%)	73 (2.1%)	1,020 (2.4%)	1,134 (2.4%)	0.019	0.287
Diabetes with Chronic Complications	47 (4.0%)	100 (2.9%)	1,845 (4.3%)	1,992 (4.2%)	0.533	<.001
Hemiplegia or Paraplegia	8 (0.7%)	13 (0.4%)	315 (0.7%)	336 (0.7%)	0.794	0.014
Renal (Kidney) Disease	14 (1.2%)	20 (0.6%)	661 (1.6%)	695 (1.5%)	0.304	<.001
Cancer (No secondary found)	16 (1.3%)	26 (0.8%)	615 (1.4%)	657 (1.4%)	0.784	<.001
Liver Disease - Moderate or Severe	*3–8	*3–8	70 (0.2%)	80 (0.2%)	0.499	0.191
Cancer (Metastatic - secondary)	*8–12	≤5	166 (0.4%)	181 (0.4%)	0.004	0.01
Mean ± SD	0.38 ± 1.14	0.19 ± 0.80	0.37 ± 1.11	0.36 ± 1.09	0.676	<.001
Median (IQR)	0 (0–0)	0 (0–0)	0 (0–0)	0 (0–0)	0.918	<.001
Charlson category					0.995	<.001
0	1,011 (85.2%)	3,181 (92.0%)	36,290 (85.3%)	40,482 (85.8%)		
1	64 (5.4%)	113 (3.3%)	2,268 (5.3%)	2,445 (5.2%)		
≥2	111 (9.4%)	165 (4.8%)	3,989 (9.4%)	4,265 (9.0%)		

IQR = interquartile range, SD = standard deviation.

**Figure 1:**
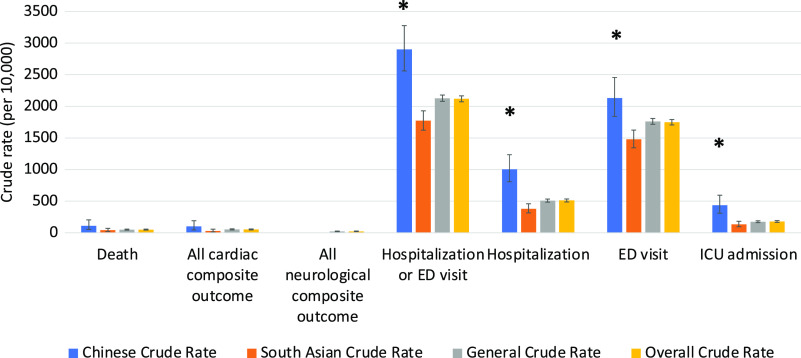
30-day crude rates by ethnic groups for under age 65 years. Error bars represent standard deviation. Asterisks indicate significant difference compared to the general crude rate. ED, emergency department; ICU, intensive care unit.

**Figure 2:**
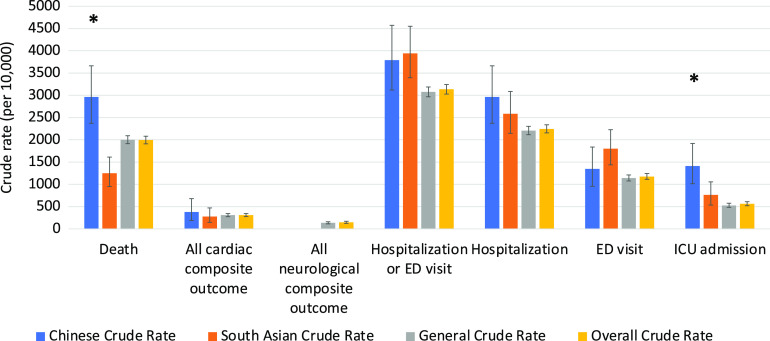
30-day crude rates by ethnic groups for age 65 years or older. Error bars represent standard deviation. Asterisks indicate significant difference compared to the general crude rate. ED, emergency department; ICU, intensive care unit.

**Figure 3:**
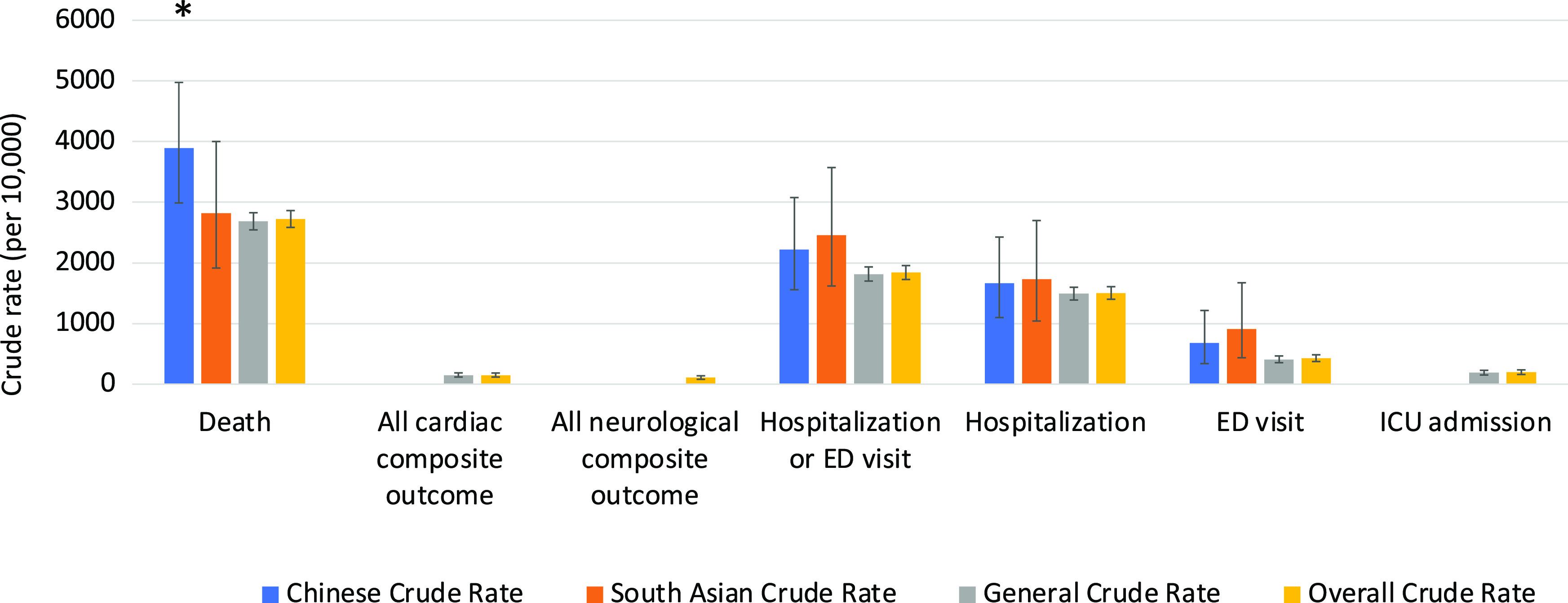
30-day crude rates by ethnic groups for patients in long-term care. Data for patients who were in long-term care in the 90-day period before being tested positive for COVID-19. Error bars represent standard deviation. Asterisks indicate significant difference compared to the general crude rate. ED, emergency department; ICU, intensive care unit.

**Figure 4:**
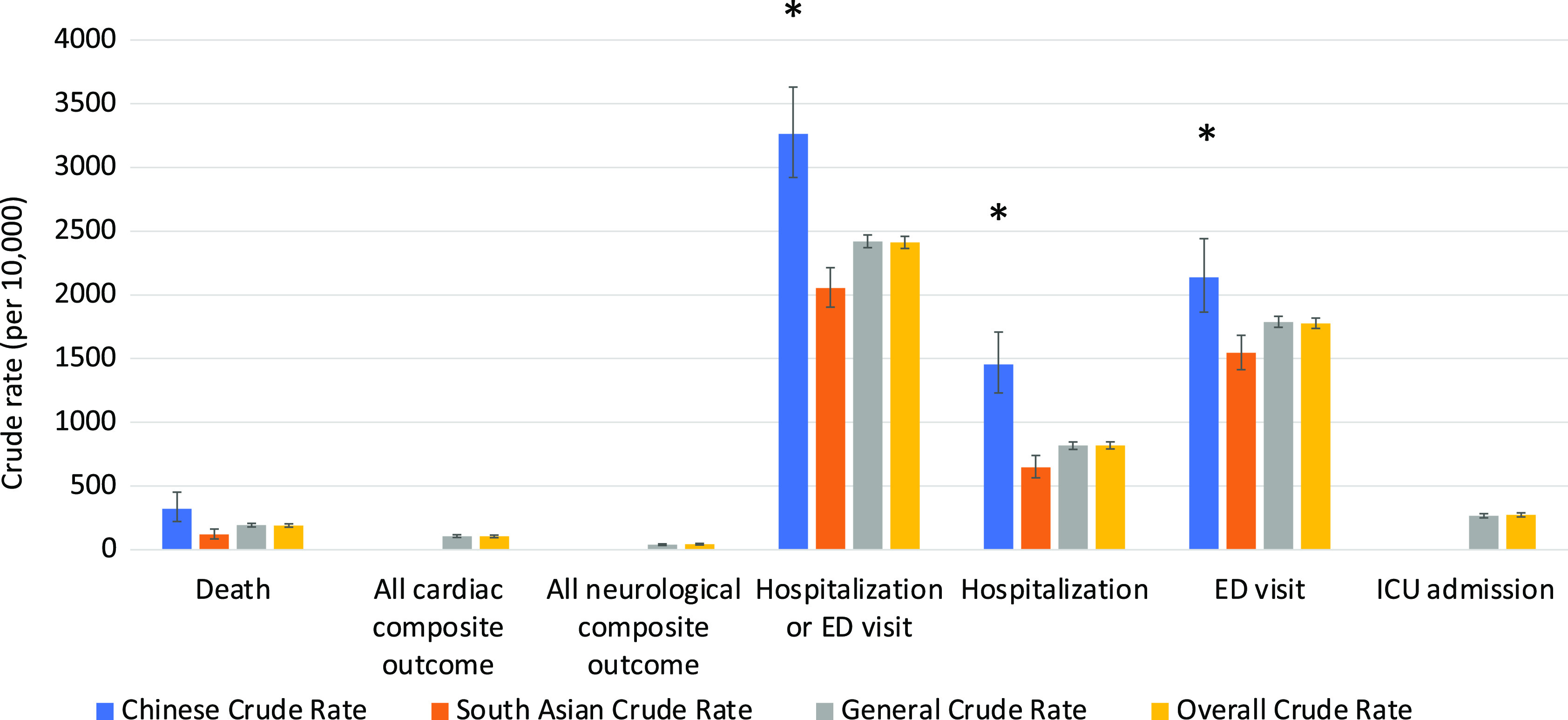
30-day crude rates by ethnic groups for patients not in long-term care. Data for patients who were not in long-term care in the 90-day period before being tested positive for COVID-19. Error bars represent standard deviation. Asterisks indicate significant difference compared to the general crude rate. ED, emergency department; ICU, intensive care unit.

**Figure 5:**
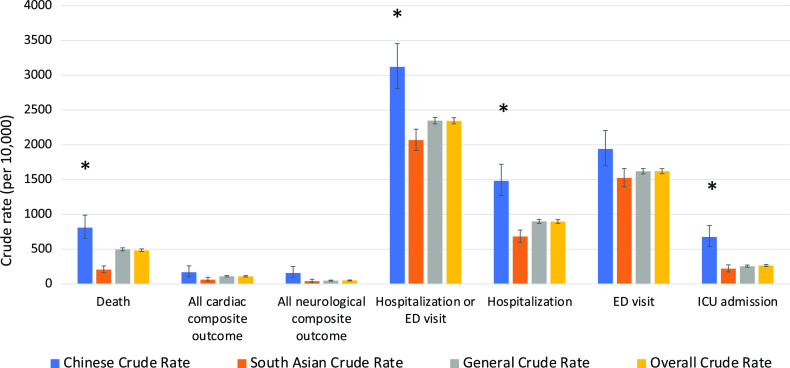
30-day crude rates for by ethnic groups. Error bars represent standard deviation. Asterisks indicate significant difference compared to the general crude rate. ED, emergency department; ICU, intensive care unit.

The clinical outcomes for all 3 cohorts are shown in Figures 1–5. Compared to the general population, emergency visits and ICU admission rates were both higher for Chinese, while they were lower in South Asians (both *p* < 0.003). These data were further dichotomized into < 65 years old (Figure 1) and for those ≥ 65 years old (Figure 2). In addition, the data were also analyzed based on those residing in long-term care facilities (Figure 3) and compared to those who were not in long-term care facilities. (Figure 4) The 30-day all-cause mortality rate was much higher for Chinese at 8.1% vs 5.0% of the general population, while it was much lower in South Asians at only 2.1% (*p* < 0.001) (Figure 5). The overall cardiac complication rate was found to be higher for Chinese compared to the general population (1.7% vs 1.1%, *p* = 0.003) and especially for those 65 years and older. The overall neurological complication rate for the general population was very low (0.5%) but was higher in Chinese (1.6%, *p* = 0.005) and tended to be lower in South Asians (0.4%, *p* > 0.05) (Figure 5). Among all the neurological complications, encephalopathy was the commonest accounting for 6.4% of hospitalized and 14.1% of Chinese admitted to ICU but it was not statistically significant compared to the general population. The 30-day crude rate for cardiac complications among Chinese was 169/10,000 (*p* = 0.069) and for the general population was 112/10,000. For neurological complications, the 30-day crude rate for Chinese was 160/10,000 (*p* < 0.001); general population was 48/10,000 (Figure 5).

For South Asians with COVID-19, the incidence of underlying diabetes was higher than the general population (19.7% vs 17.2%, *p* < 0.001), while they were lower in the following pre-morbid conditions: hypertension, heart failure, chronic obstructive pulmonary disease (COPD), dementia, and chronic kidney disease (*p* < 0.001). Only 3.2% of South Asians were in long-term care facilities compared to 12.2% in the general population (*p* < 0.001), and their incidence of hospitalization during the past 5 years from index was lower at 18.1% compared to 25.8% in the general population (*p* < 0.001) (Table 2). These differences could be due to the younger age of this cohort (Table 1). Their Charlson comorbidity index was significantly lower in incidence for heart failure, peripheral vascular disease, cerebrovascular disease, dementia, and COPD (*p* < 0.001) (Table 3). Their overall mortality rate was much lower at 2.1% compared to the general population of 5.0% (*p* < 0.001). The all cardiac complication rate was also lower at 0.6% vs 1.1% for the general population (*p* < 0.001), while the all neurological complication rate was similar to the general population (0.4% vs 0.5%, *p* = 0.526). Although their hospitalization rate was lower, there were no difference in their ICU admission rate and mechanical ventilation rate when compared to the general population (*p* > 0.05) (Figure 5). The 30-day crude rate for cardiac complications among South Asians was 40/10,000 (*p* = 0.526) compared to the general population at 112/10,000. The 30-day crude rate for neurological complications among South Asians was 64/10,000 (*p* = 0.008) compared to the general population of 48/10,000 (Figure 5).

**Figure 6:**
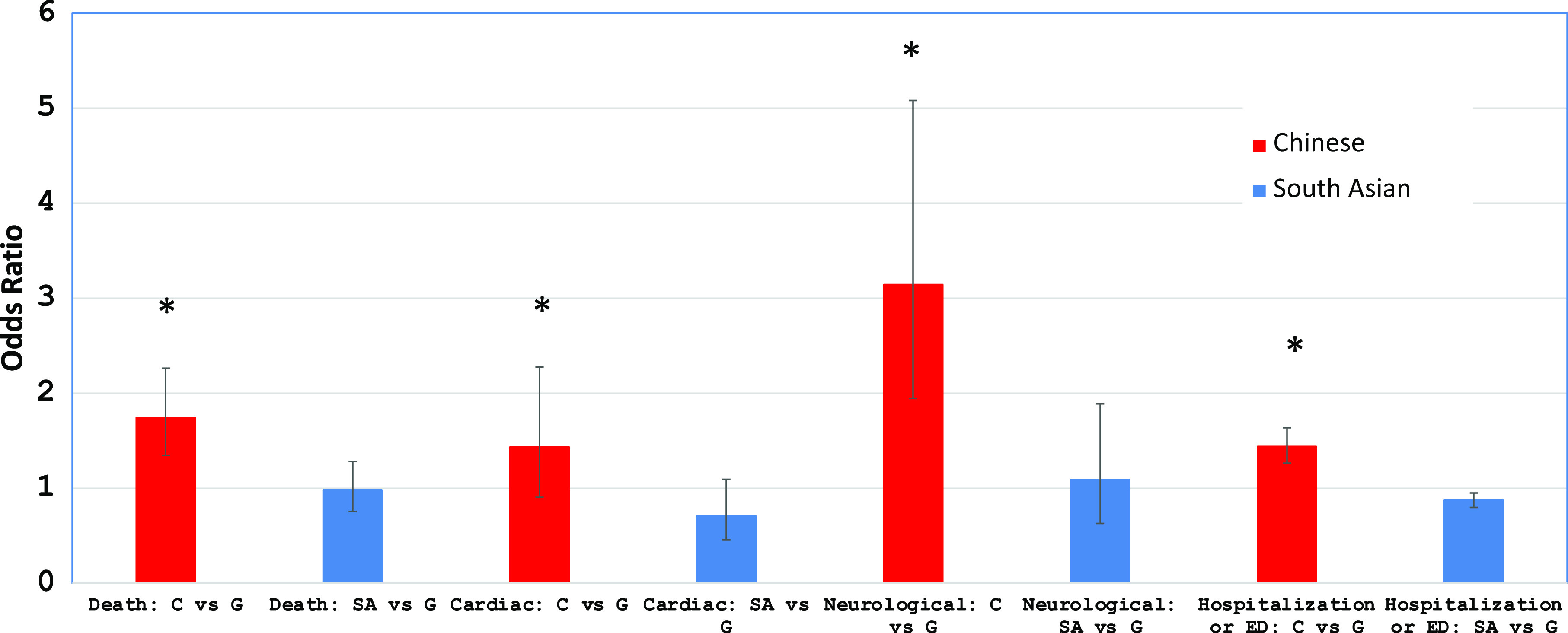
Odds ratios for 30-day outcome in Chinese and South Asian compared to the general population. Red bars represent odds ratios for Chinese compared to the general population, and blue bars represent odds ratios for South Asian compared to the general population. Error bars represent standard deviation. Asterisks indicate odds ratios that are significantly greater than 1. ED, emergency department; ICU, intensive care unit.

Multivariable logistic regression analysis demonstrated that ethnicity is a major determinant for 30 days overall mortality, cardiac, and neurological complications, hospitalization, or emergency department visits (Table 4). Chinese ethnicity, independent of their age, sex, income quintile, and long-term care placement within 90 days prior to positive COVID-19 tests were found to have higher ORs for all these outcomes, while they were lower in South Asians compared to the general population (Table 5). For Chinese, their mortality OR = 1.743; cardiac complications OR = 1.433; neurological complications OR = 3.141; and hospitalization or emergency department visits OR = 1.437. For South Asians, their mortality OR = 0.981; cardiac complications OR = 0.707; neurological complications OR = 1.088; and hospitalization or emergency department visits OR = 0.869. (Figure 6)

**Table 4:** Absolute number of events by ethnic groups

Outcomes	Chinese	South Asian	General	Overall
	N = 1,186	N = 3,459	N = 42,547	N = 47,192
Death	96 (8.1%)	71 (2.1%)	2,118 (5.0%)	2,285 (4.8%)
All cardiac composite outcome	20 (1.7%)	22 (0.6%)	476 (1.1%)	518 (1.1%)
All neurological composite outcome	19 (1.6%)	14 (0.4%)	205 (0.5%)	238 (0.5%)
Hospitalization or ED visits	370 (31.2%)	715 (20.7%)	9,981 (23.5%)	11,066 (23.4%)

ED = emergency department.

**Table 5:** Summary of odds ratios for death, cardiac complications, neurological complications, hospitalization, or emergency visits

Comparison	Odds Ratio Estimate	Lower CL	Upper CL
Death: Chinese vs General	**1.743**	**1.344**	**2.262**
Death: South Asian vs General	0.981	0.753	1.280
Cardiac: Chinese vs General	**1.433**	**0.903**	**2.274**
Cardiac: South Asian vs General	0.707	0.458	1.092
Neurological: Chinese vs General	**3.141**	**1.943**	**5.080**
Neurological: South Asian vs General	1.088	0.628	1.887
Hospitalization or ED: Chinese vs General	**1.437**	**1.263**	**1.635**
Hospitalization or ED: South Asian vs General	0.869	0.796	0.949

CL = confidence limit, ED = Emergency department visit.

Odds ratios were obtained from logistic regression. Each ethic group was compared to the general population (General). Odds ratios that are significantly different from 1 are in bold.

## Discussion

For Ontario visible minorities affected by COVID-19 during the first wave of the pandemic, Chinese were older, while South Asians were found to be relatively younger compared to the general population. This may be attributed in part to a much larger proportions of frontline health care workers and those in distribution centers are of South Asian descent and they have a lower socioeconomic status compared to the general population.^2,10^ This is further evidenced in our study indicating that only 8.2% of South Asians with COVID-19 was found to be in the highest income quintile 5, while Chinese and the general population were higher at 15.3% and 14.6%, respectively (Table 1). Based on the 2016 Canadian census,^42^ Chinese accounts for 5.7% of Ontario population, while only 2.5% of this studied group was found to have COVID-19 and this is below the expected infection rate. This could be in part an under-estimation due to the sensitivity rate of 80.2% using the last name algorithm to identify Chinese.^40^ For South Asians, they account for 8.7% of the Ontario population and 7.3% was found to have COVID-19 which is very close to the expected infection rate.^42^ Chinese when tested positive for COVID-19 were more than likely to be at long-term care facilities compared to the general population (6.4% vs 5.0%, *p* = 0.031), while it was much lower for South Asians at 1.4% (Table 3). History of heart failure (*p* < 0.004) and COPD (*p* < 0.003) were the only two baseline characteristics found to be less frequent for Chinese compared to the general population. Lower incidence of heart failure was also found among Chinese Americans in a previous study.^51^ The incidence of COPD was also found to vary by ethnicity in London, UK.^53^ Although there was no significant difference in the number of Charlson comorbidity index for Chinese, their hospitalization rates, emergency and ICU admission rates, cardiac and neurological complication rates, and overall mortality rates were much higher than the general population (Figure 5). Since many of these Chinese were residing in long-term care facilities, they were more vulnerable to be infected by COVID-19 and develop fatal complications. The overall mortality rates, hospitalization rates, ICU admission rates, and cardiac and neurological complication rates were all much higher among those 65 years or older in all three cohorts. This would indicate that age is a very important prognostication factor in patients infected with COVID-19, as reported previously.^6,15,16,26^

### Cardiac Complications

One of the biggest risk factors for severe COVID-19 and fatality from COVID-19 is underlying cardiovascular (CV) disease comorbidity.^27,40^ However, in the current study, compared to the general population, the frequencies of CV disease comorbidity such as heart failure, COPD, and asthma in Chinese were significantly lower than in the general population, while in the South Asians, these conditions were less frequent except for asthma. We assessed for acute MI, heart failure, arrhythmia in general, atrial fibrillation and flutter, myocarditis, and DVT/PE.^23^ The incidence was too low in our study to draw conclusions regarding any potential differences between groups. The heart can be affected in diverse ways by COVID-19,^31,34–38^ and mechanisms of myocardial injury^39^ in patients with COVID-19 include oxygen supply–demand imbalance, direct viral myocardial invasion,^33^ inflammation, coronary plaque rupture with acute MI, microvascular thrombosis, and adrenergic stress.^32^ The recent North American Cardiovascular COVID-19 Myocardial Infarction (NACMI) Registry^48,49^ that the primary outcome — a composite of in-hospital death, stroke, recurrent MI, or repeat unplanned revascularization —occurred in 36% of COVID-positive patients, compared with 13% of COVID-negative patients and 5% of control subjects (*p* < 0.001 relative to controls). This difference was driven largely by a high in-hospital death rate in COVID-positive patients. ST-segment elevation MI in COVID-positive patients disproportionately affects ethnic minorities (23% Hispanic and 24% Black) with diabetes, which was present in 46% of COVID-positive patients. Importantly, 23% have no culprit vessel on angiography, and this may represent different etiologies of ST-segment elevation, including microemboli, myocarditis,^33^ and stress cardiomyopathy. It is noteworthy that Asians only comprised 6% of the study population and this underscores the importance of following the cause-specific outcomes in the Asian population.

### Neurological Complications

Neurological complications of COVID-19 can be divided into two major categories: *de novo* neurological complications as a direct result of COVID-19 infections and exacerbation of pre-existing neurological conditions when patients were infected by SARS-CoV-2 virus.^7,9^ In our current study, Chinese had no significant difference in the prevalence of pre-morbid conditions based on the Charlson comorbidity index compared to the general population. This would suggest that excessive neurological complication rate of Chinese could be due to a direct result of COVID-19 infection rather than exacerbation of their pre-existing neurological conditions in addition to being older in their mean age. Among all the neurological complications, encephalopathy was the commonest accounting for 6.4% of hospitalized and 14.1% of Chinese patients admitted to ICU but it was not statistically significant compared to the general population. Encephalopathy in COVID-19 patients could be due to a combination of etiological factors: hypoxemia secondary to respiratory failure, toxic and metabolic factors secondary to acute illness and as well recently recognized inflammatory brain diseases.^4,50^ Encephalopathy of admitted COVID-19 patients in a large cohort study was associated with increased the risk of death by 5.5 times (OR = 4.01 – 7.57, *p* < 0.001).^45^ In our current study, there were very few cases of hemorrhagic^11,21^ and ischemic stroke,^20,24,30^ seizures and Parkinson’s Disease among Chinese but this could be due to the relatively small number in this cohort. The estimated incidence of stroke as a complication of COVID-19 varies between 2.5% and 5% found in various recent publications.^5,8^ In addition, there were no cases reported with encephalitis, meningitis, Guillain–Barre syndrome, and inflammatory myositis in both the Chinese and South Asians. Ongoing pathological studies will be required to examine if there are direct invasion of the SARS-CoV-2 virus in neural tissues of the central and peripheral nervous system.^4,12,47^ These clinical-pathological studies would be vital in discovering if there are different pathophysiological mechanisms in explaining the difference in neurological complications between these 3 cohorts.^46^ Although these are very rare neurological complications of COVID-19,^3,4,15,22,25,45^ we hope that with increasing number of patients in our future studies, the true incidence of these unusual neurological complications of COVID-19 among Chinese and South Asians in Ontario will be discovered.

### Limitations

We used surname algorithm to classify surnames as Chinese, South Asian, or General. The data set excludes surnames which are not unique to one of these populations such as Khan, Ahmed, or Fernandes from the South Asian list, or Lee or Young from the Chinese list. As a result, the South Asian list includes predominantly Hindu surnames and is therefore most representative of Indian surnames; Muslim surnames from Pakistan and Bangladesh are often shared with Muslim populations from other world regions and are not included in the list. These exclusions resulted in an algorithm with a high specificity (99.7% for both ethnicities) but lower sensitivity (50.4% for South Asians, 80.2% for Chinese).^40^ Another limitation is the surname algorithm cannot be used to identify Blacks and other visible minorities, and this study period covered mainly the first wave of COVID-19 in Ontario with limited data on ethnicity/race. Since we used only 30-day all-cause mortality rates, cardiac and neurological complication rates, the current study is not able to capture patients with “long-haul” COVID-19 symptoms^7,12^ even though they may have recovered from the acute illness. Another limitation relates to that hospital administrative records may not capture all complications and hence the true incidence of cardiac and neurological complications may be under-estimated in this study.^45^ We hope that with increasing number of patients in our future studies, the true incidence of these unusual neurological complications of COVID-19 among Chinese and South Asians in Ontario will be discovered.

## Conclusions

In this preliminary cohort study, using multivariable logistic regression analysis, ethnicity was found to be the most important determinant for mortality, cardiac and neurological outcomes, and hospitalization rates for those Ontarians infected by COVID-19 (Figure 6). These data have significant implications for health care policy-makers regarding resource allocation and vaccination priority^41^ in order to provide proper prevention and appropriate medical care for those Chinese and South Asians who are residing in long-term care facilities. These elderly patients are more vulnerable to be infected by COVID-19 and develop fatal complications. For South Asians infected by COVID-19, even though they were relatively younger than the general population, their overall mortality rate was still of importance particular for those 65 years and older and for those residing in long-term facilities. In addition, these findings would be of importance to Ontario public health units and health care authorities when dealing with the second and third wave of this pandemic in Ontario.

## Supplementary material

For supplementary material accompanying this paper visit http://dx.doi.org/10.1017/cjn.2021.148. click here to view supplementary material
