# Supplementary material for: COVID-19 and its Cardiac and Neurological Complications among Ontario Visible Minorities
Source: Can J Neurol Sci. 2021 Jun 24:1–10. doi: 10.1017/cjn.2021.148 (PMC8365110; doi:10.1017/cjn.2021.148)
Supplement: Supplementary file 1 [file S0317167121001487sup001.docx]

APPENDIX A: ICD-10 codes for cardiac and neurological outcomes.

# CARDIAC

| ICD 10 CA | Description |
| --- | --- |
| I21 | Acute myocardial infarction |
| I22 | Subsequent myocardial infarction |
| I50 | Heart failure |
| I4900 | Ventricular fibrillation |
| I4901 | Ventricular flutter |
| I491 | Atrial premature depolarization |
| I492 | Junctional premature depolarization |
| I493 | Ventricular premature depolarization |
| I494 | Other and unspecified premature depolarization |
| I495 | Sick sinus syndrome |
| I498 | Other specified cardiac arrhythmias |
| I499 | Cardiac arrhythmia, unspecified |
| R000 | Tachycardia, unspecified |
| R001 | Bradycardia, unspecified |
| R002 | Palpitations |
| R008 | Other and unspecified abnormalities of heart beat |
| I480 | Atrial fibrillation |
| I481 | Atrial flutter |
| I483 | Typical atrial flutter |
| I484 | Atypical atrial flutter |
| I4890 | Atrial fibrillation, unspecified |
| I4891 | Atrial flutter, unspecified |
| I495 | Sick sinus syndrome |
| I498 | Other specified cardiac arrhythmias |
| I499 | Cardiac arrhythmia, unspecified |
| I40 | Acute myocarditis |
| I41 | Myocarditis in diseases classified elsewhere |
| I802 | Phlebitis and thrombophlebitis of other deep vessels of lower extremities |
| I803 | Phlebitis and thrombophlebitis of lower extremities, unspecified |
| I801 | Phlebitis and thrombophlebitis of femoral vein |
| I828 | Embolism and thrombosis of other specified veins |
| I809 | Phlebitis and thrombophlebitis of unspecified site |
| I829 | Embolism and thrombosis of unspecified vein |
| I808 | Phlebitis and thrombophlebitis of other sites |
| I269 | Pulmonary embolism without mention of acute cor pulmonale |
| I260 | Pulmonary embolism with mention of acute cor pulmonale |

# NEUROLOGICAL

| ICD 10 CA | Description |
| --- | --- |
| I60 | Subarachnoid haemorrhage |
| I61 | Intracerebral haemorrhage |
| I63 (excluding I636) | Cerebral infarction (excluding I636 Cerebral infarction due to cerebral venous thrombosis, nonpyogenic) |
| I64 | Stroke, not specified as haemorrhage or infarction |
| H341 | Central retinal artery occlusion |
| I63 (excluding I636) | Cerebral infarction (excluding I636 Cerebral infarction due to cerebral venous thrombosis, nonpyogenic) |
| I64 | Stroke, not specified as haemorrhage or infarction |
| H341 | Central retinal artery occlusion |
| I60 | Subarachnoid haemorrhage |
| I61 | Intracerebral haemorrhage |
| G403 | Generalized idiopathic epilepsy and epileptic syndromes |
| G40 | Epilepsy  Localization-related (focal)(partial) idiopathic epilepsy and epileptic syndromes with seizures of localized onset  Localization-related (focal)(partial) symptomatic epilepsy and epileptic syndromes with simple partial seizures  Localization-related (focal)(partial) symptomatic epilepsy and epileptic syndromes with complex partial seizures  Generalized idiopathic epilepsy and epileptic syndromes  Other generalized epilepsy and epileptic syndromes  Special epileptic syndromes  Grand mal seizures, unspecified (with or without petit mal)  Petit mal, unspecified, without grand mal seizures  Other epilepsy  Epilepsy, unspecified |
| G41 | Status epilepticus  Grand mal status epilepticus  Petit mal status epilepticus  Complex partial status epilepticus  Other status epilepticus  Status epilepticus, unspecified |
| A87 | Viral meningitis |
| G039 | Meningitis, unspecified |
| A878 | Other viral meningitis |
| A879 | Viral meningitis, unspecified |
| G020 | Meningitis in viral diseases classified elsewhere |
| A85 | Other viral encephalitis, not elsewhere classified |
| A89 | Unspecified viral infection of central nervous system |
| A878 | Other viral meningitis |
| A879 | Viral meningitis, unspecified |
| F059 | Delirium, unspecified |
| G610 | Guillain-BarrT syndrome |
| M63 | Disorders of muscle in diseases classified elsewhere |
| G20 | Parkinson’s disease |
